# Non-Invasive Evaluation by the HEMOTAG^™^ Recording Device to Tailor Treatment of Acutely Decompensated Heart Failure

**DOI:** 10.70322/cvs.2025.10007

**Published:** 2025-06-27

**Authors:** Robert Chait, Fergie Ramos Tuarez, Jesus E. Pino, Dipan Uppal, David Snipelisky

**Affiliations:** 1 JFK Medical Center, University of Miami, Palm Beach Regional GME Consortium, Atlantis, FL 33462, USA; 2 Department of Cardiovascular Medicine, Mayo Clinic, Rochester, MN 55905, USA; 3 Section of Heart Failure & Transplant Medicine, Department of Cardiology, Cleveland Clinic Florida, Weston, FL 33331, USA

**Keywords:** Heart failure, Acutely decompensated heart failure (ADHF), Isovolumetric contraction time (IVCT), NT-proBNP, Cardiac time intervals (CTIs), Remote management, Wearable device, Non-invasive monitoring, Congestion

## Abstract

This study evaluated the clinical utility of the HEMOTAG^™^ recording device—A non-invasive, wearable system that measures cardiac time intervals (CTIs)—in managing patients with acutely decompensated heart failure (ADHF). The prospective, single-center study enrolled 105 patients, including those hospitalized with ADHF and a control group with non-HF-related conditions. Daily measurements of isovolumetric contraction time (IVCT), a key CTI marker, were collected using the HEMOTAG device and compared with NT-proBNP levels obtained on admission and day 3. Among ADHF patients, IVCT decreased in parallel with NT-proBNP levels, indicating volume status improvement with therapy. In contrast, the control group showed no significant change in IVCT or NT-proBNP. An IVCT ≥ 40 ms demonstrated strong sensitivity and specificity to detect ADHF (NT-proBNP ≥ 1800 pg/mL). These findings suggest that IVCT trends measured by HEMOTAG correlate with short-term treatment response in ADHF and could offer a non-invasive method to guide heart failure management. The technology demonstrated feasibility, safety, and clinical relevance, supporting its potential role in future remote management strategies.

## 1. Introduction

Heart failure (HF) is a leading cause of hospitalization, with an increasing burden on healthcare systems [1,2]. Effective treatment relies on early detection and managing cardiac congestion. N-terminal pro–B-type natriuretic peptide (NT-proBNP) is a well-established biomarker for HF severity [3,4], but non-invasive alternatives are needed.

The HEMOTAG is a wearable, non-invasive device capable of capturing cardiac time intervals (CTIs), including isovolumetric contraction time (IVCT), which may correlate with HF status [5,6]. An increase in cardiac time intervals (CTIs) has been associated with early and accurate identification of acutely decompensated heart failure (ADHF). It is likely able to provide proactive and actionable guidance in tailoring therapy. Limited data are currently available regarding the feasibility of using the HEMOTAG device in clinical practice. This study aims to evaluate the association between CTI’s as measured by the HEMOTAG device compared with NT-proBNP levels, and daily evaluation of CTIs can guide treatment for patients with ADHF to improve short-term outcomes.

## 2. Materials and Methods

The HATS OFF (Hemotag AssessmenT for Short-term Outcomes oF heart Failure) Phase I study evaluates the clinical utility of HEMOTAG-derived CTIs in detecting and managing HF decompensation in patients hospitalized with ADHF. This prospective, unblinded, single-center, non-randomized study enrolled 105 patients hospitalized with ADHF (experimental arm) and other acute medical conditions not heart failure related (control arm) over a 4-month period. The aim of this study was to assess the relationship between daily IVCTs by the HEMOTAG device and the clinical progression of ADHF. An isovolumetric contraction time (IVCT) ≥ 40 ms was used as a marker of elevated CTIs as previously validated.

### 2.1. Study Design

HATS-OFF (Hemotag AssessmenT for Short-term Outcomes oF heart Failure) is a prospective, unblinded, single-center, non-randomized observational study designed to evaluate the clinical utility of the HEMOTAG in patients with acutely decompensated heart failure (ADHF). The study assessed the relationship between HEMOTAG-derived hemodynamic parameters and established clinical markers of heart failure. Institutional review board approval was obtained prior to study initiation.

### 2.2. Study Population

Patients were initially identified using ICD-10 code I50.9 in the emergency department or on the first day of hospitalization, and patient eligibility was further assessed based on the study’s inclusion and exclusion criteria. Eligible participants provided written informed consent before study enrollment. ADHF was determined based on the hospital admission presenting with symptoms, clinical signs of heart failure, and the need for urgent intravenous diuretics.

### 2.3. Intervention and Data Collection

All enrolled patients underwent non-invasive HEMOTAG measurements daily for up to seven days or until hospital discharge, whichever occurred first. Each recording was performed using a HEMOTAG wireless sensor. The data were transmitted in real-time to a secure mobile application and cloud-based dashboard. An isovolumetric contraction time (IVCT) ≥ 40 ms was used as a marker of elevated CTIs. Additional clinical data, including baseline demographics, laboratory values (NT-ProBNP levels on Days 1 and 3), length of hospital stay, and readmission rates at 30 and 90 days, were extracted from electronic medical records (EMR) and stored in a de-identified research database.

### 2.4. Endpoints

#### 2.4.1. Primary Endpoint

To assess the association between HEMOTAG data and changes in NT-proBNP from baseline to day 3 of hospitalization.

#### 2.4.2. Secondary Endpoints

Success in getting daily HEMOTAG measurements of acceptable quality and technical fidelity on recruited patients.

### 2.5. Safety and Ethical Considerations

The HEMOTAG system is classified as a non-significant risk (NSR) device by the Food and Drug Administration (FDA) with minimal potential harm to patients. The study involved no invasive procedures, and the risks associated with electrodes were limited to minor skin irritation or discomfort. Confidentiality was maintained through secure, de-identified data storage in a locked research facility.

### 2.6. Statistical Analysis

Descriptive statistics were used to summarize baseline characteristics. The relationship between HEMOTAG measurements and NT-ProBNP changes was assessed using correlation coefficients and confusion matrix analysis. Longitudinal changes in HEMOTAG-derived parameters were analyzed using paired *t*-tests. Readmission rates were evaluated against HEMOTAG data obtained close to discharge.

### 2.7. Study Criteria

#### 2.7.1. Inclusion Criteria

1. Age greater than or equal to 22 years old.
2. An index hospital admission for ADHF based on presenting with symptoms, clinical signs of heart failure, and the need for urgent intravenous diuretics.

#### 2.7.2. Exclusion Criteria

1. Exclude enrollment by consent of a legally authorized representative.
2. Terminal condition with a life expectancy of less than 90 days.

A control cohort of hospitalized patients without a diagnosis of ADHF was utilized as a second subset within the study.

### 2.8. Device Description

**Figure 1.**
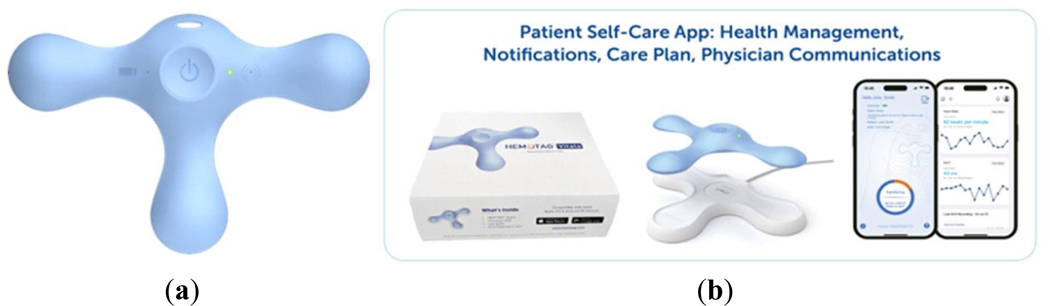
(**a**) HEMOTAG wireless sensor, (**b**) HEMOTAG Kit (Device and Mobile Application).

**Figure 2.**
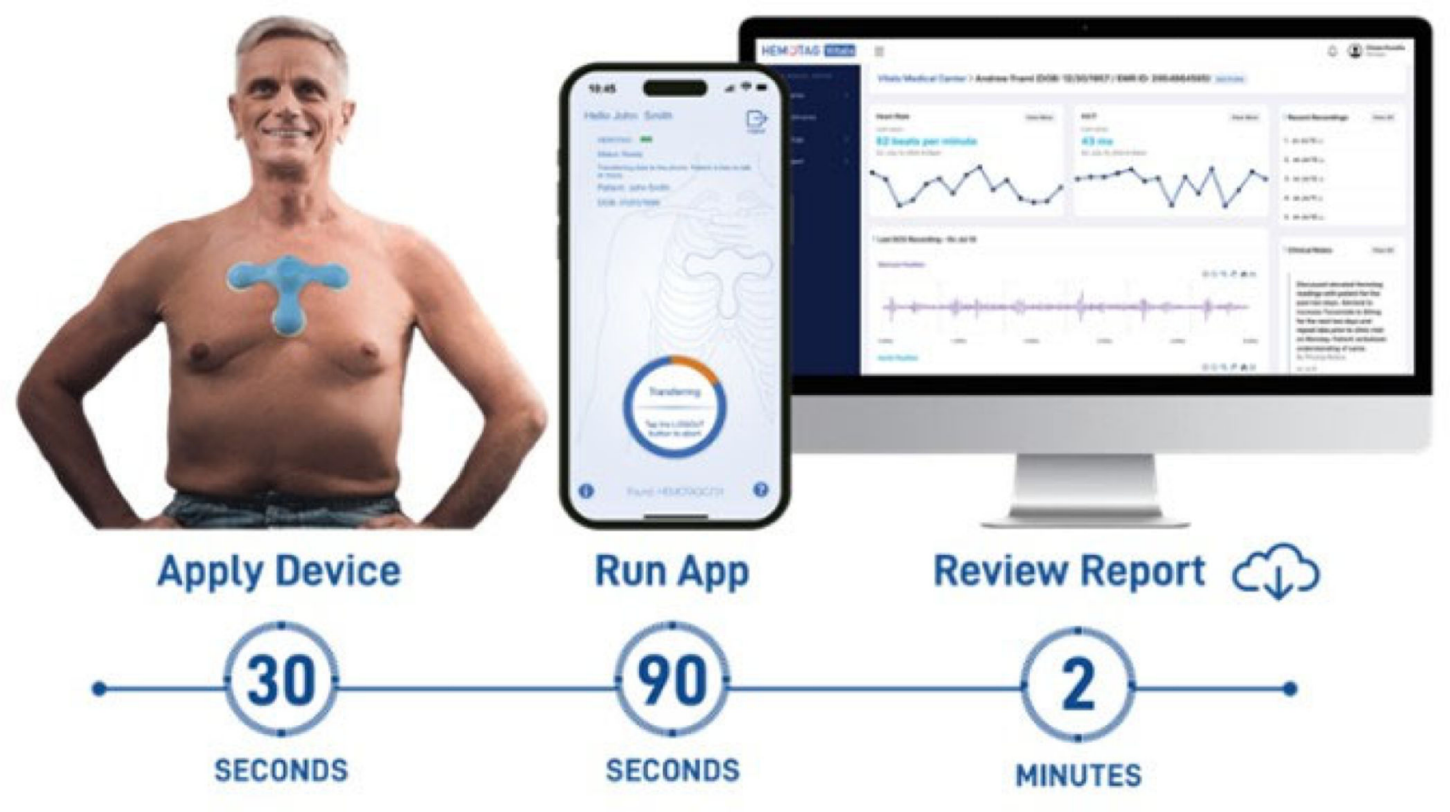
HEMOTAG Data collection process.

The HEMOTAG system consists of a sensor that is placed on the chest wall and can be used to capture 30-s measurements of electrical and heart vibrations in patients (Figure 1). The system consists of the HEMOTAG sensor, the HEMOTAG mobile application, and the HEMOTAG cloud, which together provide the end-to-end operation. Data captured by the sensor is securely transmitted via an encrypted wireless connection to the HEMOTAG Mobile App. Data is then sent through an encrypted cellular or Wi-Fi connection to the HEMOTAG central computing software, where it is temporarily stored for report generation (Figure 2). The HEMOTAG Report, which provides insights into cardiac function, is then made available to the Investigator as a PDF file.

## 3. Theory

### The Application of Cardiac Time Intervals (CTIs) to Estimate Absolute Biomarkers

CTIs are defined by the opening and closing of the aortic and mitral valve with respect to the start of the QRS complex: Aortic Valve Opening (AVO), Aortic Valve Closing (AVC), Mitral Valve Closing (MVC), and Mitral Valve Opening (MVO).

Isovolumic contraction time (IVCT = Closing of the Mitral valve to the opening of the Aortic valve = AVO − MVC). In large prospective studies, cardiac time intervals have been suggested and investigated as potential markers of hemodynamics for many years [7,8], as they are indicative of myocardial structure and function, containing physiological information about hemodynamics, namely, pulmonary artery pressures and both left ventricular systolic and left ventricular diastolic function. Multiple large studies have validated the relationship and accuracy between the CTIs and systolic and diastolic function assessed using left ventricular ejection fraction, global longitudinal strain, and global longitudinal strain rate [9]. The prognostic value of CTIs has previously been shown in various populations [10–21].

## 4. Results

### 4.1. Primary Endpoint 1—Results

ADHF was diagnosed based on hospital admission, presenting with symptoms, clinical signs, and the need for urgent intravenous diuretics. An isovolumetric contraction time (IVCT) of 40 ms was used as a marker of elevated CTIs. Eight patients were eliminated from data analysis due to unusable electrocardiogram (EKG) data or no available NT-proBNP levels. Of 97 patients who met the criteria, 44% were female, 13% were African American (Race), and 27% Hispanics (Ethnicity). The mean age and mean BMI were 63 ± 17 years and 30.5 ± 9.2 kg/m^2^, respectively (Table 1). The disease table for both groups is also shown (Table 2).

**Figure 3.**
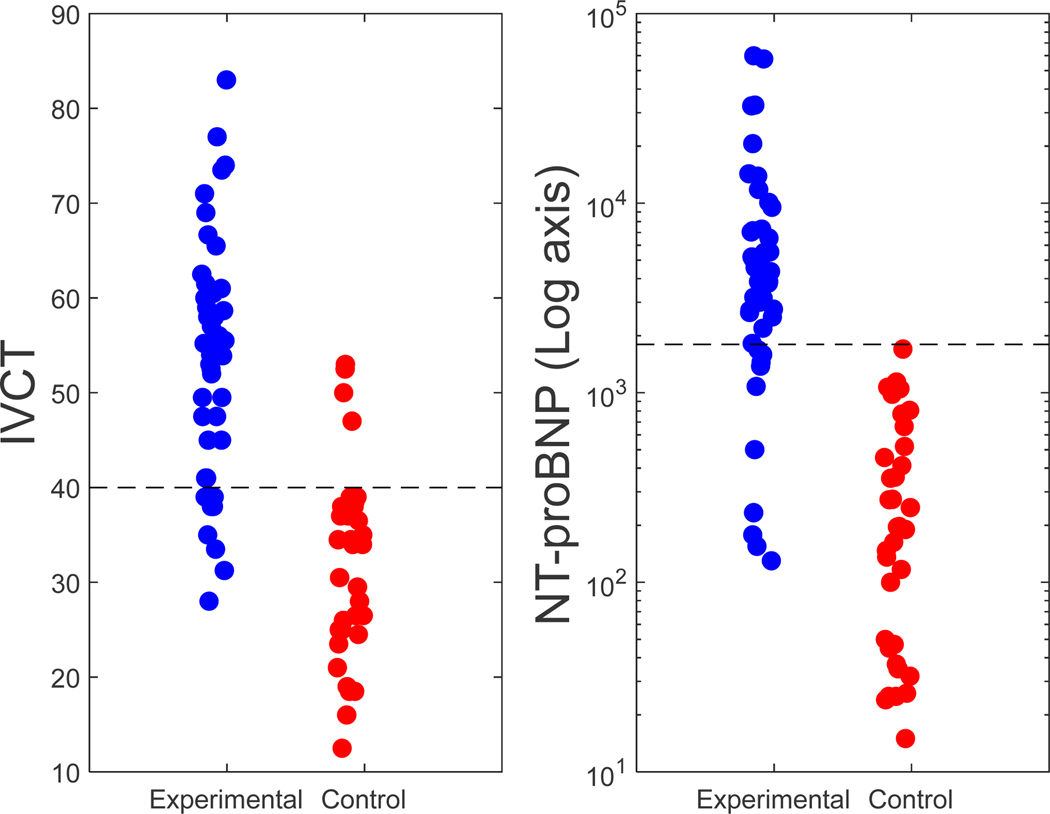
NT-ProBNP and IVCT distribution on experimental and control groups.

The mean initial NT-proBNP for the experimental arm (47 patients) was 8319 pg/mL, and the mean IVCT was 53 ms. For the control arm (50 patients), the mean initial NT-proBNP was 362 pg/mL, while the mean IVCT was 32 ms. (Figure 3).

**Figure 4.**
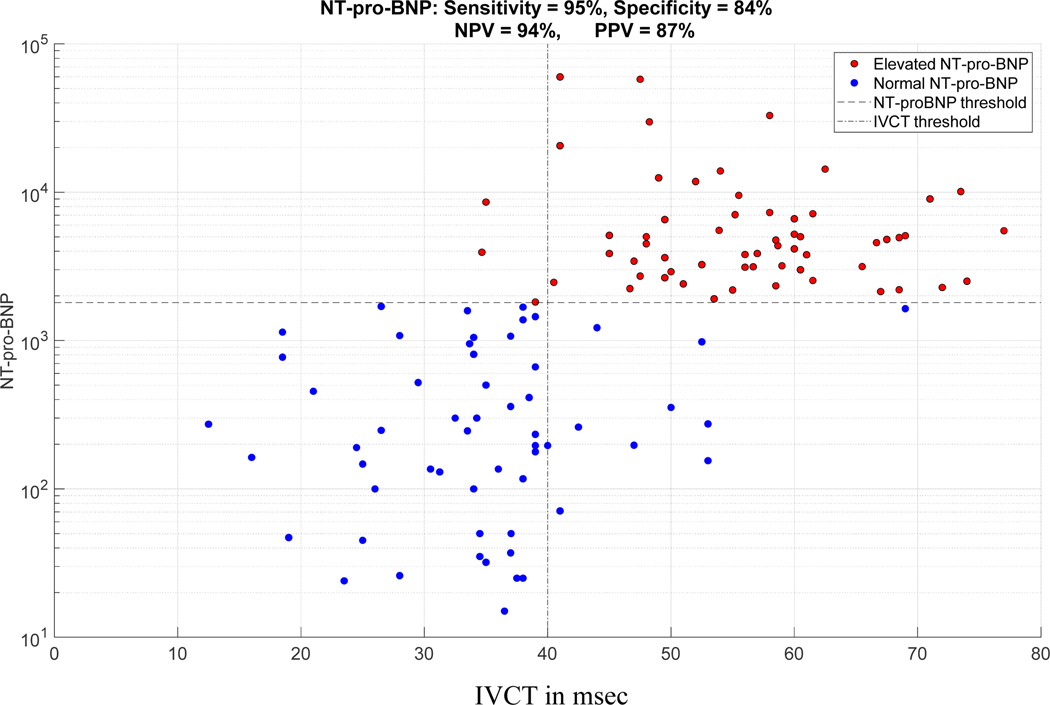
Scatter plot of IVCT *vs.* NT-proBNP.

As demonstrated in Table 3, an IVCT ≥ 40 ms showed a strong sensitivity and specificity to detect ADHF (NT-proBNP ≥ 1800 pg/mL). Figure 4 shows the scatter plot of IVCT vs NT-proBNP.

Additional analyses were performed in which NTproBNP and HEMOTAG recordings were recorded on the day following admission as well as at discharge. In this cohort, 17 patients met the eligibility criteria for the analysis (IVCT recording for a minimum of three days and NT-pro-BNP levels on the day of admission and the third day of hospitalization). Of the 17 patients who met the criteria for this analysis, 12 patients had ADHF (experimental arm) and 5 patients had an acute medical condition different than heart failure (control arm). IVCTs were recorded on admission and then daily (during hospitalization), while NT-pro-BNP was collected on admission and the 3rd day of hospitalization.

For patients with ADHF, the initial NT-pro-BNP decreased from a mean of 5515 ± 2766 pg/mL to a mean of 3801 ± 3066 pg/mL *vs.* in the control arm NT-pro-BNP went from a mean of 130 ± 72 pg/mL to a mean of 149 ± 71 pg/mL. The HEMOTAG recording device was used to track daily IVCTs in all patients. For ADHF patients, the mean IVCT went from 59 ± 10 ms (first day of hospitalization) to 49 ± 9 ms (final day of hospitalization). Conversely, the control arm did not have any significant changes in their daily IVCTs, as demonstrated by a mean of 38 ± 9 ms (first day of hospitalization) to a mean of 37 ± 3 ms (final day of hospitalization).

*T*-test suggests that the effect of the treatment the experimental group received while in the hospital affected the NT-ProBNP and IVCT values, while the untreated control group kept both values stable.

In the ADHF group: A *t*-test was conducted to determine whether the initial NT-proBNP differed from the final NT-proBNP. The analysis produced a t-statistic of *t*_Stat = 2.606, *p* = 0.024, with a critical *t*-value of *t*_critical = 2.200 at α = 0.05 (two-tailed). Since |*t*_Stat| > *t*_critical, the null hypothesis was rejected, indicating a significant difference.

A *t*-test was conducted to determine whether the initial IVCT differed from the final IVCT. The analysis produced a *t*-statistic of *t*_Stat = 2.853, *p* = 0.015, with a critical *t*-value of *t*_critical = 2.200 at α = 0.05 (two-tailed). Since |*t*_Stat| > *t*_critical, the null hypothesis was rejected, indicating a significant difference.

In the control group: A *t*-test was conducted to determine whether the initial NT-proBNP differed from the final NT-proBNP. The analysis produced a *t*-statistic of *t*_Stat = −1.631, *p* = 0.178, with a critical *t*-value of *t*_critical = 2.776 at α = 0.05 (two-tailed). Since |*t*_Stat| < *t*_critical, the null hypothesis cannot be rejected, indicating no significant difference.

A *t*-test was conducted to determine whether the initial IVCT differed from the final IVCT. The analysis produced a *t*-statistic of *t*_Stat = 0.264, *p* = 0.804, with a critical *t*-value of *t*_critical = 2.776 at α = 0.05 (two-tailed). Since |*t*_Stat| > *t*_critical, the null hypothesis cannot be rejected, indicating no significant difference.

**Figure 5.**
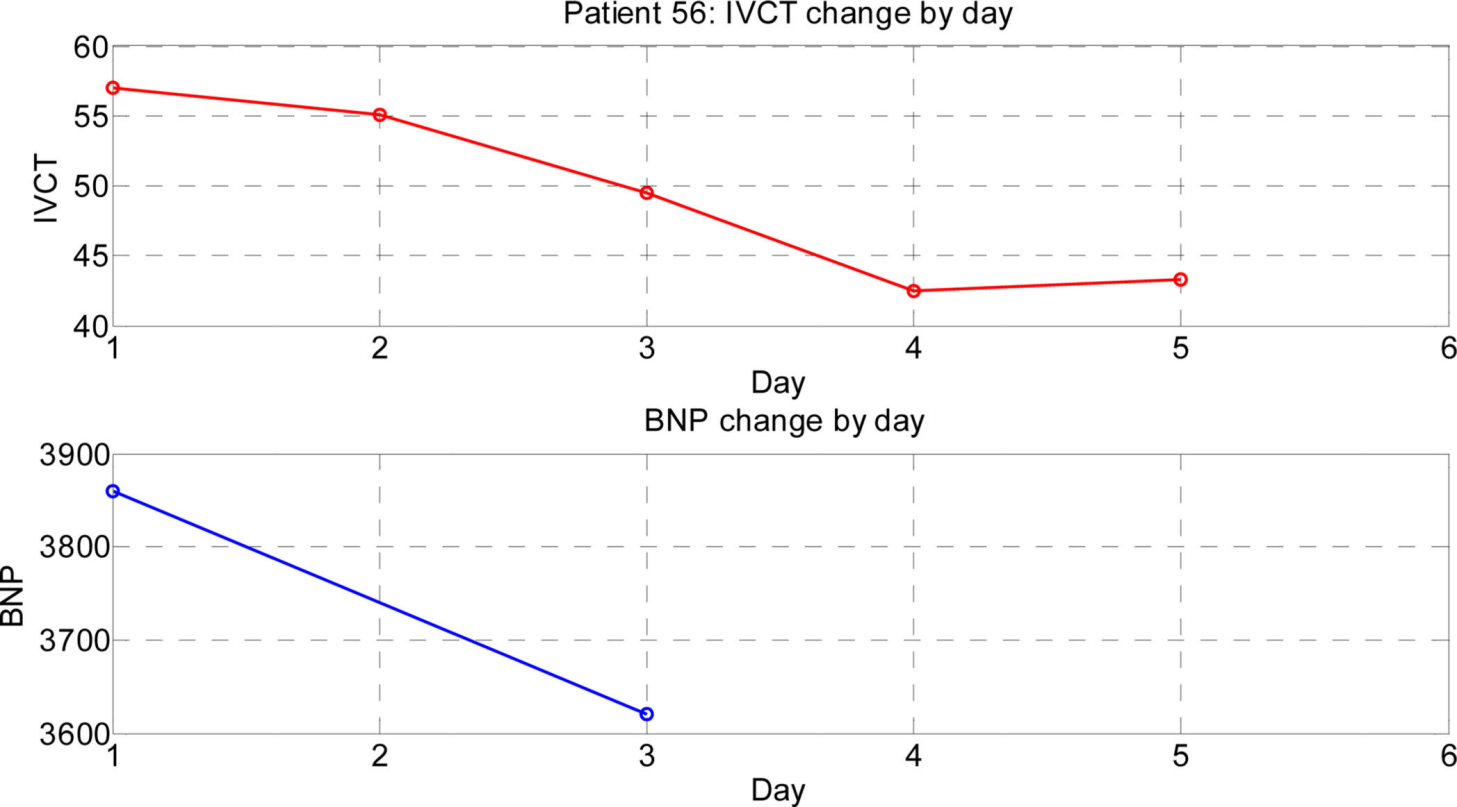
A progressive decrease in IVCT in a patient with ADHF as volume overload (congestion) decreases with daily diuretics.

Figure 5 depicts the progression of IVCTs and NT-proBNP in a subject with ADHF (experimental arm).

### 4.2. Primary Endpoint 2—Results

A total of 178 HEMOTAG recordings were sent by the 97 patients evaluated. As the experimental group of the study consisted of patients with ADHF, it was expected that most of the data would be of fair or poor quality as vibrations and electrical signals are naturally fainter and noisier. The signal quality of the data received was evaluated following the Data Quality table shown in Table 4.

The focus of this endpoint was to evaluate the success in getting daily HEMOTAG measurements of acceptable quality and technical fidelity on recruited patients and the impact of patient and medical staff training.

**Figure 6.**
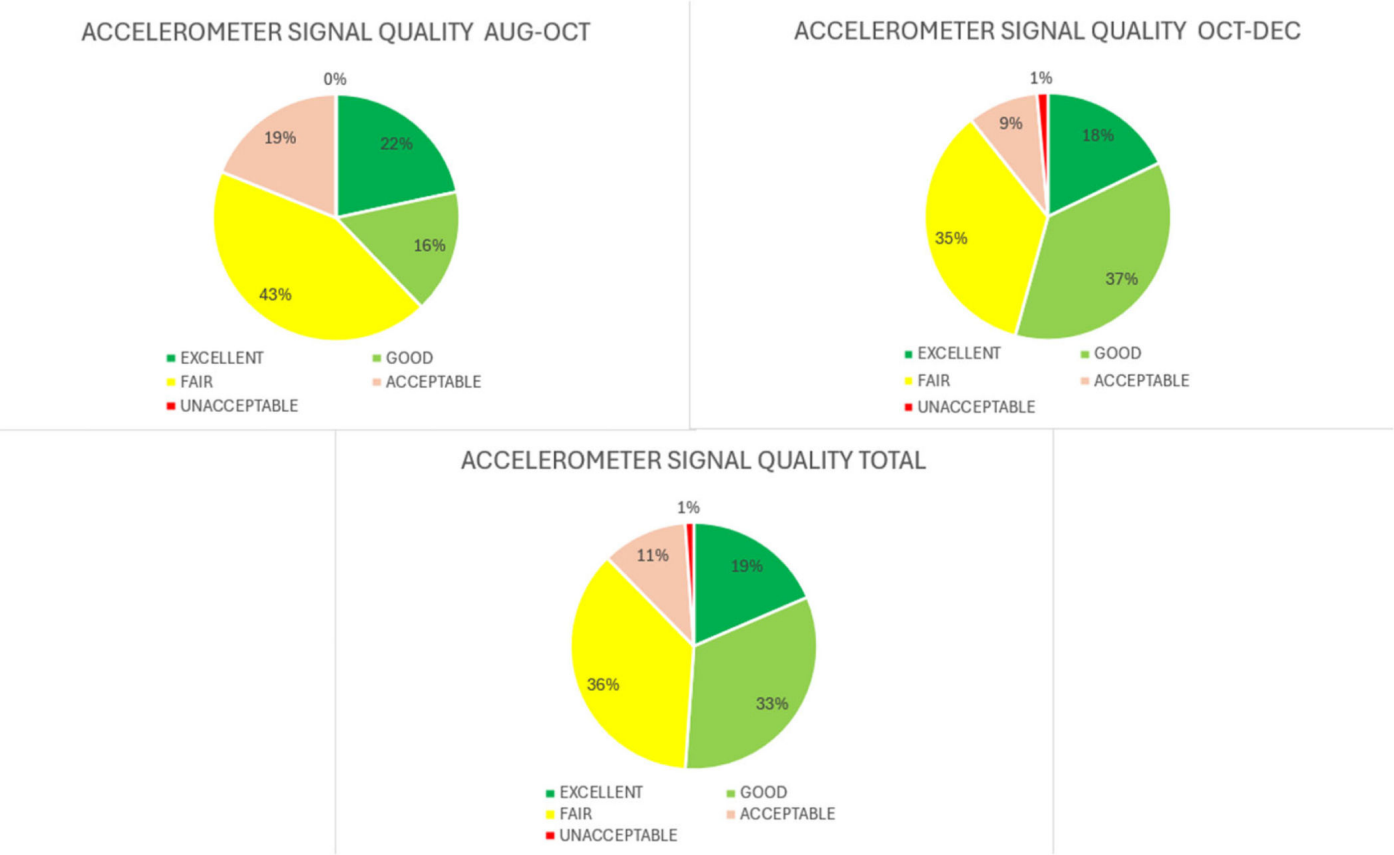
Accelerometer signal quality.

**Figure 7.**
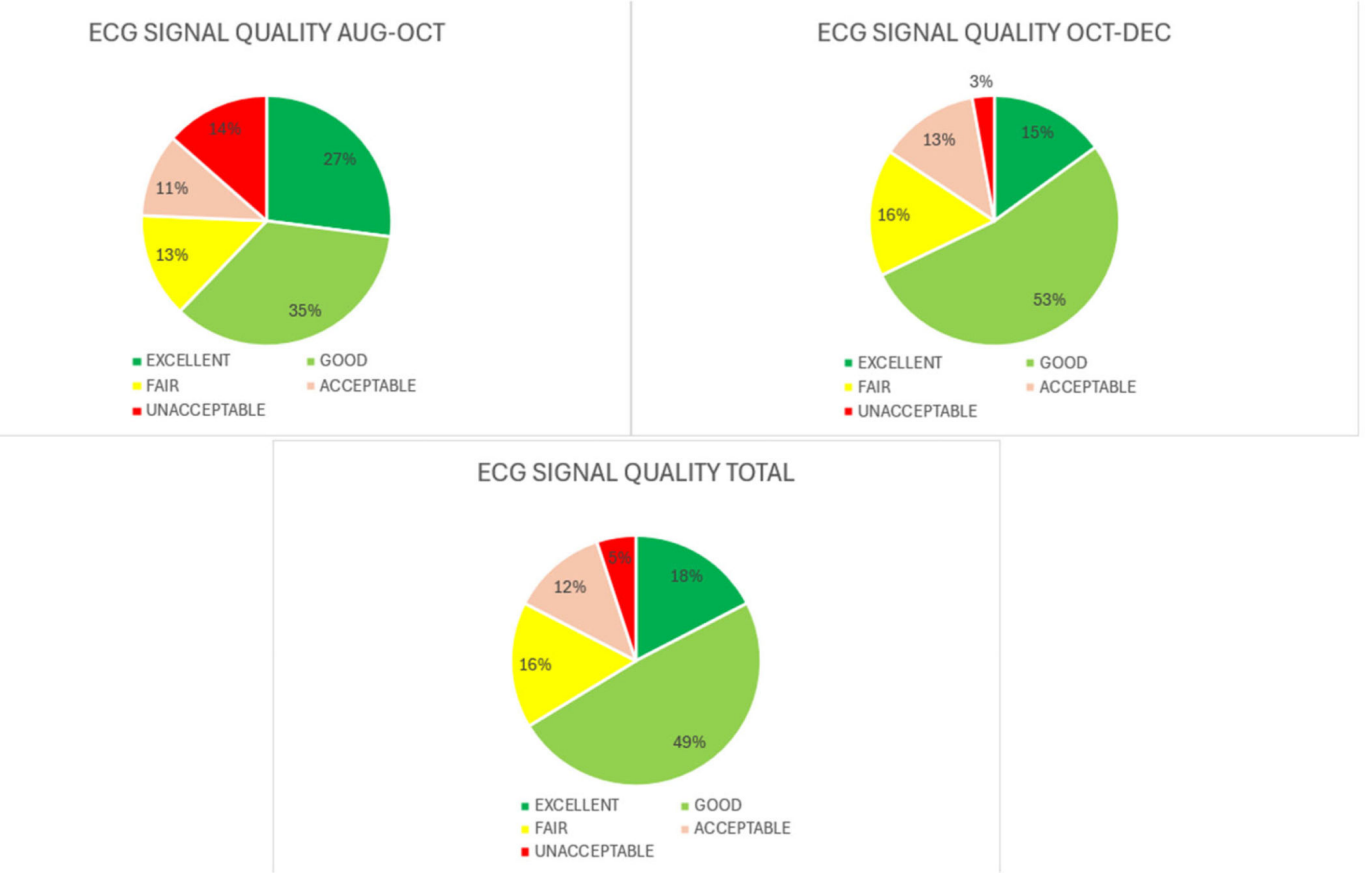
EKG/ECG signal quality.

After two months of data collection, it was found that some of the sources of data quality degradation were due to incorrect use of the device. Following the re-training of medical staff, there was an improvement in data quality for both accelerometer and EKG/ECG signals, as can be seen in Figures 6 and 7.

### 4.3. Secondary Endpoint 3—Results

The change in HEMOTAG measurements and the direction of change in HEMOTAG measurements results are summarized under primary endpoint 2.

## 5. Discussion

Isovolumetric contraction time (IVCT) and N-terminal pro–B-type natriuretic peptide (NT-proBNP) are both critical markers in heart failure (HF) assessment. However, they provide insights from different physiological mechanisms. NT-proBNP serves as a biomarker of myocardial stress, released in response to ventricular wall tension and elevated cardiac filling pressures. Meanwhile, IVCT is a mechanical parameter that reflects ventricular contractility by measuring the time interval between mitral valve closure and aortic valve opening. This study demonstrated that daily IVCTs, as measured by the HEMOTAG recording device, can predict the progression and response to treatment (*i.e.*, volume status) of patients with ADHF.

The relationship between IVCT and NT-proBNP is complex yet interconnected. Prolonged IVCT has been observed in patients with both heart failure with reduced ejection fraction (HFrEF) and heart failure with preserved ejection fraction (HFpEF) and is often associated with increased myocardial stiffness and delayed ventricular contraction [22,23]. As myocardial strain increases due to higher left ventricular filling pressures, NT-proBNP secretion also rises [24]. This suggests that IVCT prolongation may be an indicator of myocardial stress [5], providing a potential non-invasive alternative to NT-proBNP.

While existing literature has established independent associations between these two parameters, direct studies evaluating their correlation remain limited. Our study explores this relationship further, particularly in the context of non-invasive, real-time management.

The HEMOTAG provides a non-invasive method for measuring cardiac time intervals (CTIs), including IVCT, by integrating heart vibrations and electrocardiogram (EKG) data. This study aimed to assess the agreement between HEMOTAG-derived measurements and NT-proBNP levels, which could yield important clinical insights. By evaluating IVCT, HEMOTAG could detect subclinical myocardial stress. This would enable intervention and better prognostic stratification for heart failure patients. Additionally, IVCT measurement with HEMOTAG could be performed non-invasively at the bedside or remotely, unlike NT-proBNP, which requires laboratory testing. From a practical standpoint, this could reduce hospital readmissions for heart failure and hopefully would translate into cost savings.

Invasive devices, such as the CardioMEMS Heart Failure System [24], have demonstrated that hemodynamic measurements allow more effective heart failure management, leading to fewer hospitalizations by identifying worsening cardiac dynamics prior to symptom onset. In view of the risk and cost of invasive devices and the growing number of patients needing convenient access to personalized disease management, there is a clear need for non-invasive methods that can accurately manage heart failure in the clinic and at home [6]. HEMOTAG may be able to provide similar value in a non-invasive fashion.

Study limitations exist. Our study includes a relatively small population size and is a single-center study. Additionally, this study is observational, and all patients in the study arm already had a diagnosis of ADHF. Therefore, it is difficult to understand the efficacy of the HEMOTAG device in predicting the acute onset of decompensated heart failure. Although these limitations exist, the findings of this study help support additional research to further understand the utility of this device in predicting and preventing the onset of acutely decompensated heart failure.

The results of this study indicate a strong relationship between IVCT and NT-proBNP as there was a parallel decrease in HEMOTAG measured IVCT to NT-ProBNP levels as the ADHF patients were treated. This suggests that daily IVCTs by the HEMOTAG recording device could predict the progression and response to treatment (*i.e.*, volume status) of patients with ADHF.

## 6. Conclusions

This study demonstrates a significant relationship between the NT-proBNP biomarker and IVCT as measured by the non-invasive HEMOTAG device. It provides support for the use of the HEMOTAG device as a potential non-invasive tool in the identification of acutely decompensated heart failure. While NT-proBNP remains a standard biomarker, HEMOTAG’s ability to non-invasively manage a heart failure patient using IVCT may provide additional prognostic value. Future studies should aim to validate whether changes in IVCT correspond to changes in hemodynamics over time, paving the way for a more comprehensive approach to heart failure monitoring.

## Figures and Tables

**Table 1.** Patient Demographics.

Measure	N	Mean	Standard Deviation	Median	Min	Max
Age (years)	97	62.99	16.89	64	22	91
Weight (kg)	97	90.27	33.16	81.50	43	259
BP systolic	97	128.92	41.39	128	84	182
BP diastolic	97	73.16	9.95	73	53	98
Height (cm)	97	170.66	11.14	170	149	205
BMI	97	30.54	9.24	28.71	16.80	69.53

**Table 2.** Clinical Characteristics and Cardiac Comorbidities.

Disease States	Number of Patients Experimental	Number of Patients Control
1. Fluid-management Problems	43	5
2. Heart Failure (HF):	43	9
2.1. Systolic HF	18	6
2.2. Diastolic HF	22	3
2.3. Systolic and Diastolic HF	3	0
3. Coronary Artery Disease (CAD)	25	12
4. Hypertension (HTN)	43	34
5. Pulmonary hypertension (PHTN)	14	0
6. Diabetes:	26	15
6.1. Type I	0	4
6.2. Type II	26	11
7. Lung Disease	11	5
8. Mitral Valve Disease:		
8.1. Mitral Regurgitation	14	5
8.1.1. Mild	6	4
8.1.2. Moderate	6	1
8.1.3. Severe	2	0
9. Aortic Valve Disease:		
9.1. Aortic Regurgitation	3	3
9.1.1. Mild	1	3
9.1.2. Moderate	1	0
9.1.3. Severe	1	0
9.2. Aortic Stenosis	3	2
9.2.1. Mild	1	1
9.2.2. Moderate	2	1
9.2.3. Severe	0	0
10. Tricuspid Valve Disease:		
10.1. Tricuspid Regurgitation	14	2
10.1.1. Mild	7	2
10.1.2. Moderate	4	0
10.1.3. Severe	3	0
11. Cardiomyopathy	44	12
12. Left Ventricular Hypertrophy (LVH)	1	0
13. NYHA HF	44	11
13.1. Class 1	1	6
13.2. Class 2	16	4
13.3. Class 3	23	1
13.4. Class 4	4	0
14. Sleep Apnea	12	6
15. Angina	18	10
16. Previous Myocardial Infraction	24	5
17. Pacemaker	7	2
18. ICD	10	1
19. Undergone Chemotherapy	2	0
20. VTE	1	2
21. AFIB	25	4
22. COPD	10	6
23. CKD	14	3

**Table 3.** HEMOTAG efficacy to detect ADHF (NT-proBNP ≥ 1800 pg/mL).

Statistical Measure	Result
Sensitivity	95
Specificity	84
Negative Predictive value	94
Positive predictive value	87
The area under the curve	0.92
R	0.58

An IVCT ≥ 40 ms recorded by HEMOTAG has high sensitivity and specificity to detect ADHF. In this analysis, all patients (except the control group) have clinical evidence of ADHF. An NT-pro BNP ≥ 1800 pg/mL was used as an objective surrogate of ADHF.

**Table 4.** HEMOTAG Data Quality Table.

QUALITY LEVEL	HEMOTAG EKG/ECG	HEMOTAG Heart Vibrational Signals
EXCELLENT	QRS complex is clearly defined and has a very low noise level.	The onset and offset of the signal are clearly identifiable, and the noise is very low compared to the main signal.
GOOD	QRS complex defined, but some noise or wandering effect is present.	The onset and offset of the signal are identifiable, but some noise is present in the system on some or all of the heartbeats.
FAIR	QRS complex has a noticeable noise/wandering effect that may make it difficult to find the onset of the QRS complex at some heartbeats.	The signal vibration level and the noise are comparable in amplitude, making it difficult to clearly define the onset and offset of the signal for some heartbeats.
ACCEPTABLE	QRS complex has a noticeable noise/wandering effect that may make it difficult to find the onset of the QRS complex at most heartbeats.	The signal vibration level and the noise are comparable in amplitude, making it difficult to clearly define the onset and offset of the signal for most heartbeats. A heart sound may be completely absent from the data.
UNACCEPTABLE	EKG signal is unreadable.	Accelerometer signal is unreadable.

## Data Availability

The data is protected as per the guidelines of SBIR data protection from for a period of not less than 20 years with the protection period beginning at the time of the Phase award (R44HL145941 – 06/01/2019).
